# Utilization of Mirror Visual Feedback for Upper Limb Function in Poststroke Patients: A Systematic Review and Meta-Analysis

**DOI:** 10.3390/vision7040075

**Published:** 2023-11-15

**Authors:** Hyunjoong Kim, Eunsang Lee, Jihye Jung, Seungwon Lee

**Affiliations:** 1 Neuromusculoskeletal Science Laboratory, 306 Jangsin-ro, Gwangju 62287, Republic of Korea; hyun-joongkim@nmslab.org; 2 Department of Physical Therapy, Gwangju Health University, 73 Bungmun-daero 419, Gwangju 62287, Republic of Korea; eslee@ghu.ac.kr; 3 Institute of SMART Rehabilitation, Sahmyook University, 815 Hwarang-ro, Seoul 01795, Republic of Korea; jihye3752@gmail.com; 4 Department of Physical Therapy, Sahmyook University, 815 Hwarang-ro, Seoul 01795, Republic of Korea

**Keywords:** virtual reality, mild cognitive impairment, cognitive function, rehabilitation

## Abstract

Mirror visual feedback (MVF), a noninvasive treatment method, is attracting attention as a possibility to promote the recovery of upper limb function in stroke patients. However, the cognitive effects of this therapy have received limited attention in the existing literature. To address this gap, we conducted a systematic review and meta-analysis to investigate the relationship between upper limb function and cognition in stroke patients and to evaluate the effect of MVF on improving upper limb function. A comprehensive search was performed on the Embase, MEDLINE, and PubMed databases to identify original articles and clinical studies published between 2013 and 2022. Qualitative analysis was performed using the Cochrane Risk of Bias tool, and in the quantitative analysis, a random-effects model was used as the effect model, and standard mean difference (SMD) was used as the effect measure. Eight studies that met the inclusion criteria were entered in the analysis. Data extraction included an assessment tool for upper extremity function. Results of the quantitative analysis demonstrate that MVF was effective in improving upper extremity function in stroke patients (SMD = 0.94, 95% CI 0.69 to 1.20). In conclusion, this systematic review and meta-analysis provides evidence supporting the effectiveness of MVF in improving upper limb function in stroke patients. However, further studies are needed to investigate the cognitive effects of MVF and elucidate the underlying mechanisms.

## 1. Introduction

Stroke is a prevalent neurological condition that often leads to long-term disabilities, particularly affecting upper limb function [1,2,3,4,5]. In the realm of stroke recovery, an integrated approach to rehabilitation is essential, melding physical, occupational, speech, and cognitive therapy to facilitate functional restoration [6]. As part of this multifaceted effort, therapeutic interventions often target cognitive deficits and strive to activate the brain’s plasticity, including synaptic reinforcement and long-term potentiation, which are vital for neurological rehabilitation [7,8]. Amidst the evolving landscape of therapeutic strategies to bolster motor recovery, mirror visual feedback (MVF) has gained traction as an innovative and effective modality to retrain the brain and improve motor functions in stroke survivors [9,10,11,12,13,14,15]. MVF involves the use of a mirror to provide visual feedback, creating an illusion of movement in the affected limb by reflecting the movement of the unaffected limb. This technique has shown promise in facilitating motor recovery and promoting neural plasticity in stroke patients [16,17].

While the efficacy of MVF in enhancing motor outcomes has been widely investigated, its potential impact on cognitive function in stroke patients remains a topic of interest and uncertainty [16]. Cognitive impairments are prevalent in stroke survivors and can significantly impact their overall recovery, functional independence, and quality of life [18,19,20]. Exploring the potential cognitive benefits of MVF is crucial for optimizing stroke rehabilitation interventions and improving patient outcomes [16,18].

The aim of this systematic review and meta-analysis is to comprehensively examine the utilization of MVF for upper limb function in stroke patients, with a specific focus on its effects on cognition. By synthesizing and analyzing available evidence from relevant studies, we aim to provide a comprehensive overview of the current knowledge in this area and shed light on the potential cognitive advantages of MVF in stroke rehabilitation.

## 2. Materials and Methods

### 2.1. Protocol and Registration

This systematic review and meta-analysis protocol was developed according to the Preferred Reporting Items for Systematic Reviews and Meta-Analyses (PRISMA) guidelines. The protocol was registered in the international prospective register of systematic reviews (PROSPERO) with the registration number CRD42022360633.

### 2.2. Search Strategy

A comprehensive literature search was conducted in electronic databases, including the Excerpta Medica (Embase), Medical Literature Analysis and Retrieval System Online (MEDLINE), and PubMed databases. The search strategy was designed to identify relevant studies published from inception to the present. The search terms were organized based on Medical Subject Headings. The search terms are a combination of keywords related to “post-stroke”, “mirror visual feedback”, “cognitive function”, and “randomized controlled trial”. The search strategy was adjusted based on the specific requirements of each database. The final search expression used was ((stroke OR post-stroke OR cerebrovascular accident) AND (mirror therapy OR mirror visual feedback) AND (cognition OR cognitive function) AND (randomized controlled trial)).

### 2.3. Study Selection

For the study selection process, two independent reviewers carefully examined the titles and abstracts of the identified studies to assess their suitability for inclusion in the review. The selection criteria was based on the PICOSD framework, which stands for Population, Intervention, Comparison, Outcome, and Study Design.

#### 2.3.1. Inclusion Criteria

The inclusion criteria for this systematic review and meta-analysis are as follows:

1. Participants: Only studies involving stroke patients are considered eligible for inclusion. The participants’ characteristics, such as age, gender, and stroke severity, are taken into account.
2. Intervention: Only interventional studies that evaluate the use of MVF for upper limb rehabilitation are included. MVF therapy involves the use of mirrors to provide visual feedback to stroke patients during rehabilitation exercises.
3. Outcomes: All outcome variables related to cognition are included in the review. This could include measures of cognitive function, such as attention, memory, executive function, and language. If three or more identical outcome variables are reported across studies, they are synthesized for quantitative analysis.
4. Type of studies: Only published randomized controlled trials (RCTs) are included.

#### 2.3.2. Exclusion Criteria

The exclusion criteria for this review are as follows. Non-English studies: studies published in languages other than English were excluded due to limitations in language proficiency among the review team. Publication date: studies published before 2013 were excluded to ensure the inclusion of the most recent research in the field. MVF as control group: studies that include MVF as the control group were excluded. This is to ensure a clear distinction between the MVF intervention and other forms of therapy.

### 2.4. Data Extraction

The studies identified through the electronic database searches were exported to Microsoft Excel (Microsoft, Redmond, Washington, DC, USA) for further analysis. To ensure the integrity of the data, a thorough process was implemented to identify and remove duplicate studies from the dataset. Duplicate studies can arise from multiple sources, such as different databases or search strategies yielding overlapping results. By carefully examining the titles, authors, and other relevant information, the reviewers ensured that each study included in the subsequent stages of the review was unique and distinct.

Data extraction, a critical step in the systematic review and meta-analysis process, was performed by two reviewers independently. A standardized data extraction form was utilized to capture various study characteristics. These characteristics may include authors, publication year, study design, sample size, participant demographics, details of the MVF intervention, outcome measures employed, and the reported results. In cases where there were discrepancies between the two reviewers during the data extraction process, a consensus was reached through discussion. This discussion allows for clarification and resolution of any discrepancies or differing interpretations of the extracted data. If required, a third reviewer was consulted to provide further input and assist in reaching a consensus.

### 2.5. Quality Assessment

The Cochrane Risk of Bias tool was employed to assess the quality of the included studies, ensuring the originality and integrity of the content [21]. Two independent reviewers evaluated the risk of bias in each study based on specific domains, including random sequence generation, allocation concealment, blinding of participants and personnel, blinding of outcome assessment, incomplete outcome data, selective reporting, and other biases. The risk of bias for each domain was categorized as low, high, or unclear based on the reviewers’ assessments. This rigorous evaluation process aims to provide an objective and comprehensive assessment of the quality and reliability of the included studies without compromising the integrity of the research.

The Cochrane Risk of Bias tool was employed to assess the quality of the included studies in the systematic review and meta-analysis. This tool is widely recognized and accepted as a standardized approach to evaluate the risk of bias in randomized controlled trials (RCTs) and other types of studies. Two independent reviewers evaluated the risk of bias for each included study based on specific domains outlined in the Cochrane Risk of Bias tool. These domains include the following:

1. Random sequence generation: The method used to generate the random sequence allocation was assessed to determine whether it was conducted adequately and with minimal risk of bias.
2. Allocation concealment: The process of concealing the allocation sequence from the researchers or participants was evaluated to assess whether it effectively prevented selection bias.
3. Blinding of participants and personnel: The extent to which participants and personnel involved in the study were blinded to the intervention being evaluated was assessed. Blinding helps to minimize performance bias and the influence of knowledge or expectations on study outcomes.
4. Blinding of outcome assessment: The assessment of outcomes by independent evaluators who are blinded to the intervention was evaluated to minimize detection bias.
5. Incomplete outcome data: The extent to which data were missing or incomplete and whether this could introduce bias in the results was assessed. Additionally, the appropriateness of any statistical methods used to handle missing data was considered.
6. Selective reporting: The completeness of outcome reporting was assessed to identify any potential bias due to selective reporting of outcomes.
7. Other biases: Any other potential sources of bias not covered by the above domains, such as conflicts of interest or funding sources, were evaluated.

The risk of bias for each domain was categorized as low, high, or unclear based on the assessments made by the reviewers. By conducting independent assessments by two reviewers, the researchers aimed to evaluate the internal validity of the included studies and identify any potential biases that may affect the reliability and validity of the findings.

### 2.6. Data Synthesis and Meta-Analysis

The data synthesis and analysis of the selected papers were conducted using RevMan 5.4, a software developed by The Cochrane Collaboration based in Oxford, England. In cases where three or more papers reported the same variables, they were included in the quantitative and meta-analyses. To address the inherent heterogeneity among the included studies, a random-effects model was employed for the analysis, allowing for more robust and conservative estimations. The effect sizes were calculated using the standard mean difference (SMD), which facilitated the comparison of outcomes across different studies.

The heterogeneity between studies was assessed using both the Chi-square test and the I^2^ test. The I^2^ statistic provides insights into the proportion of total variation across studies due to heterogeneity. Interpretation of the I^2^ results indicates that values exceeding 75% represent high heterogeneity, while values below 40% indicate low heterogeneity [22]. Additionally, to explore the potential publication bias, a funnel plot, which is a graphical tool, was employed, especially when the number of included papers exceeded 10 [23]. The funnel plot allows for a visual assessment of the symmetry of the distribution of effect sizes, aiding in the evaluation of potential publication bias.

### 2.7. Ethical Considerations

As this study is a systematic review and meta-analysis based on published literature, ethical approval is not required.

## 3. Results

### 3.1. Literature Search and Characteristics of the Included Trials

**Figure 1:**
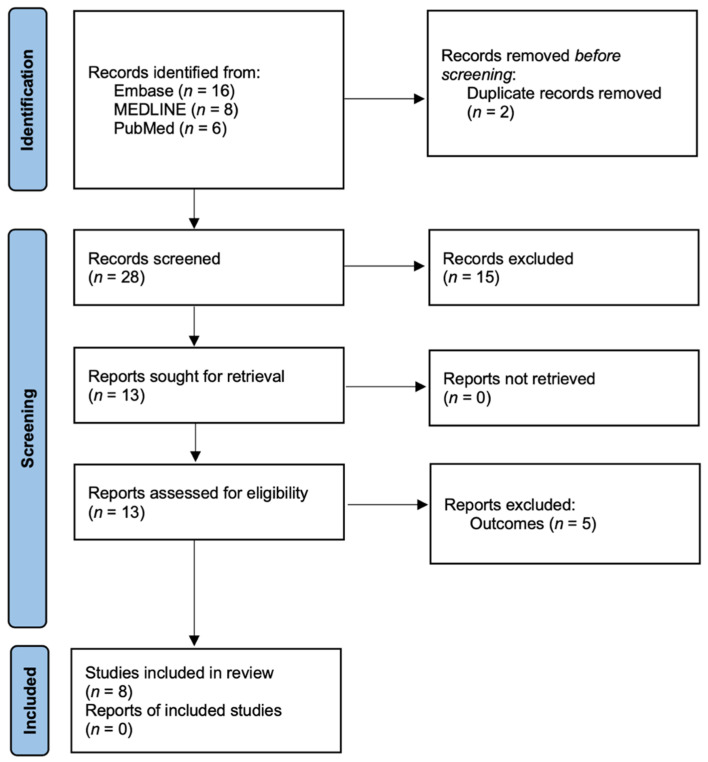
Preferred Reporting Items for Systematic Reviews and Meta-Analyses flow diagram.

Figure 1 illustrates the flowchart depicting the study selection process. The initial search conducted in three international electronic databases yielded a total of 30 papers. After removing two duplicate studies using Microsoft Excel, the titles and abstracts of the remaining 28 papers were reviewed and screened. Based on the screening process, 15 cases were excluded as they did not meet the predefined inclusion criteria. The inclusion criteria may have involved factors such as study design, population, intervention, outcome measures, or other specific criteria deemed relevant to the research question. Subsequently, the full texts of the remaining 13 studies were obtained and thoroughly examined to determine their eligibility for inclusion. Upon careful examination, an additional five studies were excluded based on specific exclusion criteria, such as language, study design, or relevance to the research question. Finally, after a thorough screening and selection process, eight studies were deemed to meet the inclusion criteria and were selected for further analysis. These eight studies underwent data extraction and subsequent synthesis and analysis as part of the systematic review and meta-analysis [12,24,25,26,27,28,29,30].

### 3.2. Assessment of Methodological Risk of Bias

The assessment evaluated specific domains for each included study, including random sequence generation, allocation concealment, blinding of participants and personnel, blinding of outcome assessment, incomplete outcome data, selective reporting, and other biases. For random sequence generation, all eight studies were classified as low-risk, indicating that the method used to generate the random sequence allocation was adequately conducted with minimal risk of bias. Similarly, all eight studies were classified as low-risk for allocation concealment, suggesting that the process of concealing the allocation sequence was effective in preventing selection bias. In terms of the blinding of participants and personnel, three studies were classified as low-risk, indicating that participants and personnel involved in the study were adequately blinded to the intervention. However, three studies were classified as uncertain-risk, indicating a lack of clear information regarding blinding, and two studies were classified as high-risk, suggesting a high potential for performance bias. For the blinding of outcome assessments, three studies were classified as low-risk, indicating that outcome assessors were adequately blinded. Four studies were classified as uncertain risk, indicating incomplete reporting or insufficient information regarding blinding, while one study was classified as high-risk, suggesting a high potential for detection bias. Six studies were classified as low-risk for incomplete outcome data, indicating a low risk of bias due to missing or incomplete data. However, two studies were classified as high-risk, suggesting a high potential for bias resulting from missing data. Regarding selective reporting, five studies were classified as low-risk, indicating a low risk of bias due to selective reporting of outcomes. Three studies were classified as uncertain-risk, suggesting incomplete reporting or insufficient information. In terms of other biases, three studies were classified as low-risk, indicating a low risk of bias from other sources. However, five studies were classified as uncertain risk, suggesting the presence of potential biases that were not adequately addressed or reported. This classification was based on previous studies [31] that indicated the importance of preregistration to reduce other biases.

### 3.3. Mirror Visual Feedback in Patients with Mild Poststroke

Table 1 provides an overview of the characteristics of eight RCTs conducted on a total of 256 stroke patients. These trials examined the effects of interventions involving both general and mixed MVF. The primary focus of assessment in these trials was on upper limb function, which was evaluated using the ABILHAND questionnaire [27], Fugl-Meyer assessment (FMA) [12,24,26,28,29,30], and manual function test (MFT) [25] as outcome measures. In addition, the intervention period was variously included as 3 [12,29], 4 [30], 5 [25,26], 6 [24,27], and 8 weeks [28].

### 3.4. Effectiveness of Mirror Visual Feedback in Treating Poststroke Patients

**Figure 2:**
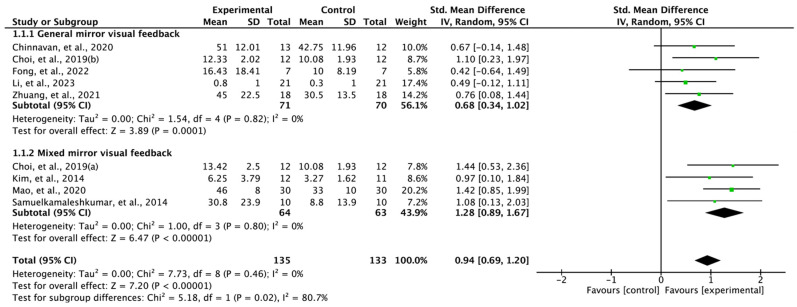
Forest plot studying the effect of mirror visual feedback on upper limb function. Choi et al., 2019(a) [25]: gesture recognition mirror therapy; Choi et al., 2019(b) [25]: mirror therapy. (Refs. are cited [12,24,25,26,27,28,29,30] in figure).

The combined results from eight RCTs confirmed that MVF produced significant positive improvements in upper limb function in stroke patients: SMD = 0.94, 95% confidence interval [CI]: 0.69 to 1.20, heterogeneity: χ^2^ = 7.73, df = 8, I^2^ = 0%, and overall effect: Z = 7.20, *p* < 0.00001. Subgroup analysis showed significant improvement in both general MVF (SMD = 0.68, 95% CI: 0.34 to 1.02, heterogeneity: χ^2^ = 1.54, df = 4, I^2^ = 0%, and overall effect: Z = 3.89, *p* = 0.0001) and mixed MVF (SMD = 1.28, 95% CI: 0.89 to 1.67, heterogeneity: χ^2^ = 1.00, df = 3, I^2^ = 0%, and overall effect: Z = 6.47, *p* < 0.00001) (Figure 2).

### 3.5. Publication Bias

For the meta-analysis in this review, a total of eight studies were included based on the predetermined eligibility criteria. As recommended by the Cochrane Review [32], no publication bias analysis was performed due to the relatively small number of studies included in the synthesis.

## 4. Discussion

Our systematic review and meta-analysis are a comprehensive synthesis of the effects of MVF on poststroke patients, with a particular emphasis on cognition. While the majority of studies have primarily examined upper limb function, this review also integrates a synthesis of upper limb function and describes its impact on cognition through neurological mechanisms.

In the study involving 256 stroke patients [12,24,25,26,27,28,29,30], the results indicate a significant improvement in upper limb function with MVF (SMD = 0.94, 95% CI: 0.69 to 1.20). The analysis was conducted based on the characteristics of MVF, classifying it into general-type and mixed-type. The general MVF demonstrated a moderate effect size (SMD = 0.68, 95% CI: 0.34 to 1.02) [12,24,25,30], while the mixed MVF exhibited a large effect size (SMD = 1.28, 95% CI: 0.89 to 1.67) [25,26,28].

When reviewing the results in conjunction with previous studies, the meta-analysis revealed high heterogeneity and a moderate effect size (SMD = 0.51) in relation to the synthesis of upper limb motor function among stroke patients [33]. In another meta-analysis [34], a synthesis of 32 cases indicated that the effect of MVF on upper limb function might be insignificant based on the protocol. In a meta-analysis that synthesized data from 17 RCTs on gait speed (SMD = 1.04), mobility (SMD = 0.46), and motor recovery (SMD = 0.47), all of them were analyzed to have a large effect size [35]. Based on the aforementioned previous studies [33,34,35], it has been established that MVF leads to positive improvements in motor function. Therefore, it is appropriate to conclude that the results of the meta-analysis synthesized in this systematic review exhibit low heterogeneity and a high effect size, further supporting the efficacy of MVF.

Furthermore, in our review, we conducted subgroup analyses to examine the distinctive characteristics of general MVF and mixed MVF. The findings revealed a larger effect size for mixed MVF (SMD = 1.28) compared with general MVF (SMD = 0.68). Interestingly, similar results were reported in a previous study, where 10 randomized controlled trials were synthesized. The study also found that incorporating MVF alongside the existing rehabilitation program was effective [36]. Although several studies have investigated the combined effects of MVF [37,38], there is limited understanding of the neurological mechanisms underlying these effects. However, as MVF plays a crucial role in the reconstruction of sensory circuits [39], it can provide partial compensation in this regard.

MVF is reported to have potential effects on cognition indirectly rather than directly. From the concept of mirror neurons, these neurons are characterized by firing when an individual performs an action and when they observe someone else performing the same action [40]. It is believed that mirror neurons contribute to the imitation and learning of motor skills [41]. In this context, MVF has the potential to enhance cognitive functions related to motor performance. We tried to interpret it in terms of cognition to be consistent with the purpose of this systematic review. From a neurological point of view, it can be explained through changes in action potentials in MVF [16]. In the interaction between actual feedback and expected feedback induced by MVF, and between visual feedback and kinesthetic feedback for actually felt perception, it was confirmed that excitability was changed in the following cortices: superior parietal lobe, superior posterior parietal cortex, posterior cingulate cortex, and ipsilateral lateral sulcus [42,43,44,45]. Furthermore, when considering cortex function, it has been reported that MVF, when combined with motor imagery, induces additional changes and increases in cortical excitability [46,47]. However, it should be noted that several studies have also reported a lack of activation in the ipsilateral primary motor cortex (M1) [48,49,50].

Research findings indicate that cognitive processes [51], including attention, working memory, and executive functions, play a role in the planning and execution of complex upper limb movements. Similarly, difficulties in upper limb function can have a detrimental effect on cognitive abilities, particularly those related to motor planning and coordination. Therefore, it is important to acknowledge the need to recognize changes in cognitive function as an essential task in the future in order to understand the neurological basis for improvement in upper limb function, considering the limited neuroplastic effects of MVF in neurorehabilitation.

The strength of this systematic review and meta-analysis lies in their ability to demonstrate the consistent advantages of MVF in improving upper limb function, as supported by numerous previous studies. Additionally, it confirms that combining MVF with existing neurorehabilitation programs can offer even greater benefits. Furthermore, the review provides clear evidence, from a cognitive perspective, of the improvement in upper limb function through underlying neurological mechanisms. 

Our systematic review and meta-analysis had the following limitations. As there were numerous review articles on MVF already reported, we used only three representative databases to avoid duplicated literature, which led to a relatively small number of RCTs being synthesized. While this could mean that the studies included were of lower heterogeneity and higher quality, it may limit generalizability. Furthermore, we intended to synthesize the effects on cognitive function, but there were not enough studies to do so; therefore, the effects were only explained as being indirectly mediated through improvements in upper limb function. This underscores the need for further research in this area. Methodologically, categorization based on the timing of onset (acute vs. chronic) could be additionally described in future studies regarding the neuroplastic changes induced by mirror feedback in neurorehabilitation.

## 5. Conclusions

MVF offers beneficial effects for functional improvement in poststroke patients with reduced upper limb function. Moreover, when combined with existing neurorehabilitation, additional MVF leads to greater functional improvement compared with when MVF is performed alone.

## Figures and Tables

**Table 1:** Characteristics of included trials.

Study	Sample Size	Duration	Upper Limb Function	Intervention (Therapeutic Intensity)	Authors’ Conclusion
Chinnavan et al., 2020 [24]	EG = 13 CG = 12	6 weeks	FMA	EG = Mirror therapy (3 times a week, 45 min per session. For the first 30 min, therapy is applied to the affected upper limb, and the remaining 15 min are applied to the unaffected upper limb only) CG = Conventional therapy (Mobilization, reaching, grasping and dexterity 3 times a week, 45 min per session)	Integrating conventional therapy with mirror therapy proves beneficial for upper limb motor function recovery in hemiplegic patients.
Choi et al., 2019 [25]	EG1 = 12 EG2 = 12 CG = 12	5 weeks	MFT	EG1 = Gesture recognition mirror therapy (30 min 3 times a week 3D-motion-input-device-based mirror therapy) EG2 = Mirror therapy (30 min per session, 3 times a week general mirror therapy) CG = Control (Sham 3 times a week, 30 min per session)	Mirror therapy utilizing gesture recognition devices enhances functionality, reduces neck discomfort, and improves life quality in chronic stroke survivors.
Fong et al., 2022 [12]	EG = 7 CG = 7	3 weeks	FMA	EG = Mirror therapy (30 min task-specific training 4 times a week) CG = Sham (Sham using a covered mirror 4 times a week, 30 min per session)	Mirror visual feedback is more effective than a covered mirror in mitigating spatial neglect symptoms, without a marked advantage over bilateral transparent glass movements.
Kim et al., 2014 [26]	EG = 12 CG = 11	5 weeks	FMA subcategory; hand	Both groups received conventional rehabilitation training for 60 min per session, 5 times a week. EG = FES with MT (5 times a week, 30 min per session) CG = FES without MT (5 times a week, 30 min per session)	The application of functional electrical stimulation alongside mirror therapy in poststroke care significantly advances upper extremity motor function.
Li et al., 2023 [27]	EG = 21 CG = 21	6 weeks	ABILHAND questionnaire	Both groups offer home programs 5 times a week EG = Bilateral robotic priming combined with mirror therapy 3 times a week, 90 min per session CG = Bilateral robotic priming combined with bilateral arm training 3 times a week, 90 min per session	Enhanced motor improvement in upper limbs is evident when mirror therapy is combined with bilateral robotic priming, with effects persisting for three months.
Mao et al., 2020 [28]	EG = 30 CG = 30	8 weeks	FMA	CT; Upper limb rehabilitation training (5 times a week, 60 min per session) and Schulte Grid training (5 times a week, 30 min per session) EG = MNSP (5 times a week, 20 min per session) plus CT CG = CT	A synergy of mirror-neuron-system-based training with conventional rehabilitation practices improves motor and cognitive functions in stroke-affected upper extremities.
Samuelkamaleshkumar et al., 2014 [29]	EG = 10 CG = 10	3 weeks	FMA	5 times a week, 6 h per session PMRP EG = Mirror therapy (5 times a week, 1 h per session) plus PMRP CG = PMRP	The employment of mirror therapy in conjunction with bilateral arm training and graded tasks yields better motor performance in stroke-induced paretic upper limbs compared with conventional therapy.
Zhuang et al., 2021 [30]	EG = 18 CG = 18	4 weeks	FMA	EG = Associated mirror therapy CG = Control	Our research indicates that associated mirror therapy is an effective strategy for facilitating motor recovery and daily functioning in individuals with stroke-affected limbs.

CG, control group; EG, experimental group; FES, functional electrical stimulation; FMA, Fugl-Meyer assessment; MFT, manual function test; MNSP, mirror neuron system training; MT, mirror therapy; PMRP, patient-specific multidisciplinary rehabilitation program.

## Data Availability

Data are contained within the article.
